# Oral lichen planus in a patient treated with anti‐CD20 monoclonal antibodies

**DOI:** 10.1111/ddg.15777

**Published:** 2025-06-04

**Authors:** Willy Chan, Alexander Nast, Gabriela Poch, Kamran Ghoreschi

**Affiliations:** ^1^ Department of Dermatology Venereology and Allergology Charité – Universitätsmedizin Berlin corporate member of Freie Universität Berlin and Humboldt‐Universität zu Berlin Berlin Germany

Dear Editors,

A 53‐year‐old female patient presented in our dermatological outpatient clinic with painful, bilateral, whitish, reticular lesions on the oral buccal mucosa consistent with Wickham´s striae (Figure 1). The patient also complained about itch and increased sensitivity of the genital mucosa as well as itch and desquamation of the scalp. The genital mucosa was free from skin alterations, the scalp showed flat scaly patches. The nails were not affected. The family history was positive for psoriasis. Prior to the manifestation of the skin symptoms the patient had received treatment with ocrelizumab, an anti‐CD20 monoclonal antibody for her multiple sclerosis. Ocrelizumab (600 mg) had been administered at six‐months intervals (June 2022, December 2022, July 2023). First skin symptoms were noticed 4 weeks after the third infusion.

**FIGURE 1:**
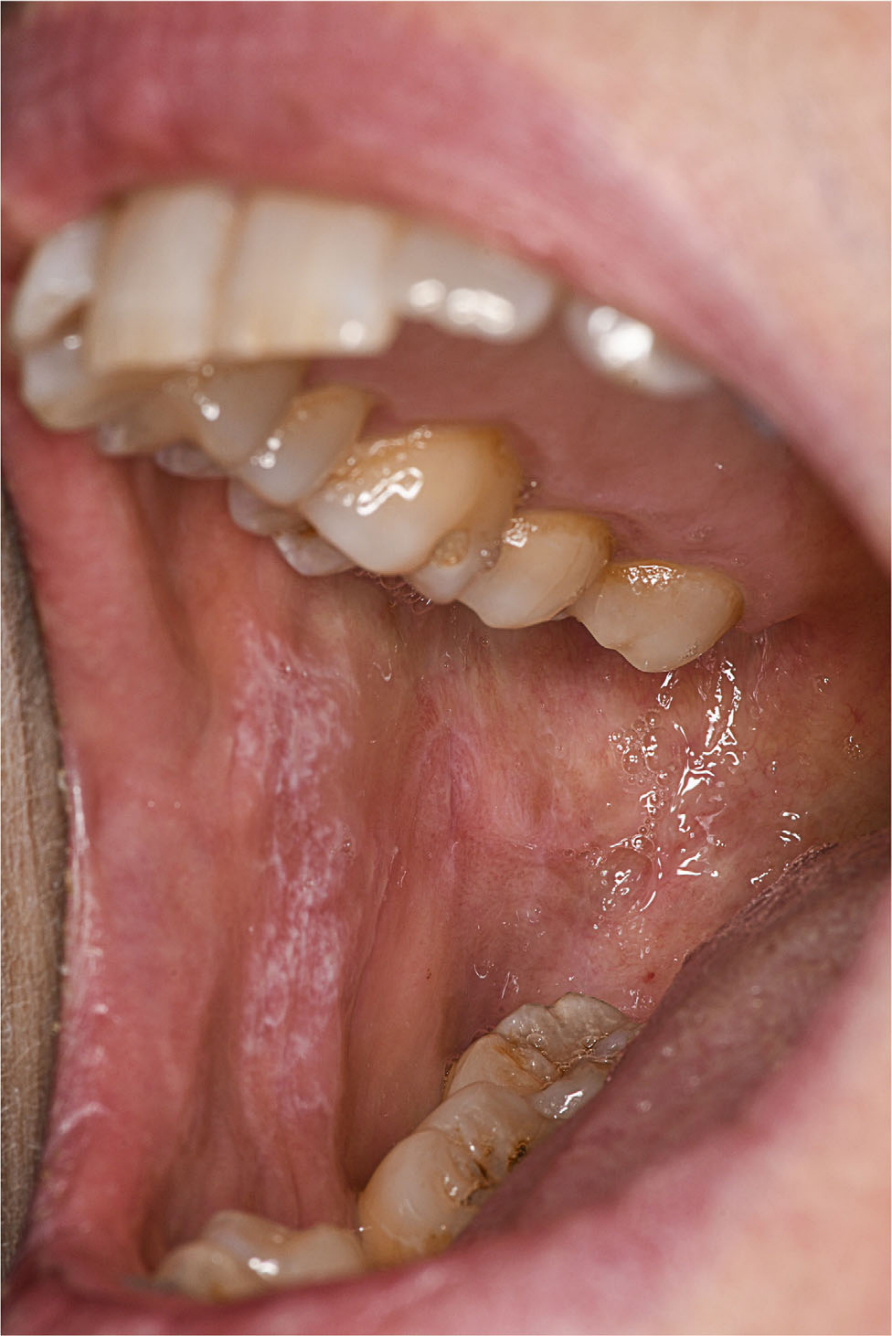
Whitish, reticular lesions on the oral buccal mucosa consistent with Wickham's striae.

We performed a punch biopsy from the buccal mucosa. Histopathological examination showed a band like lymphocytic infiltrate at the dermoepidermal junction with basal cell vacuolization, indicative for an interface dermatitis and typical for lichen planus.

Ocrelizumab infusions were discontinued. For the treatment of lichen planus topical treatment with glucocorticosteroids was initiated: Betamethasone mouthwash was continuously used for the oral lesions, methylprednisolone creme was applied to the genital mucosa and clobetasol shampoo was applied to the scalp. With this treatment, the skin manifestations were markedly reduced but a complete remission has yet to be achieved. Regarding the multiple sclerosis the patient is currently stable and not receiving any treatment. A continuation of treatment with anti‐CD20 antibodies is not planned. Future therapeutic options include dimethyl fumarate or sphingosine‐1‐phosphate receptor modulators.

To evaluate a possible causality or coincidence between the anti‐CD20 therapy and the manifestation of lichen planus, we searched for previously published cases. We were only able to identify one case of a lichenoid reaction due to ocrelizumab reported in the literature so far.^1^ We were able to identify two further reported cases associated with the anti‐CD20 antibodies rituximab^2^, ^3^ and one case with obinutuzumab.^4^ In contrast to our patient, in all reported cases patients showed full remission after topical corticosteroids and discontinuation of anti‐CD20 therapy. Besides lichenoid reactions, there have been reports of psoriasiform lesions (predominantly palmoplantar pustulosis) associated with anti‐CD20 therapy.^5^, ^6^, ^7^, ^8^, ^9^ Since lichen planus and psoriasis are both T‐cell mediated diseases, a hyperactive T‐cell response due to the depletion of B cells has been hypothesized. However, the pathomechanism is not yet fully understood.^3^ Interestingly, there are also reports of rituximab as a successful treatment for lichen planus.^10^, ^11^, ^12^ While the induction of a lichenoid reaction as an adverse drug reaction is a common pattern in dermatology, it has rarely been reported as a result of anti‐CD20 therapy. Given the large number of patients that have received anti‐CD20 antibodies over the past three decades, a total of five published cases highlights the rarity of this side effect. With this publication, we intend to draw further attention to anti‐CD20 antibodies as a possible trigger of lichenoid inflammation.

## CONFLICT OF INTEREST STATEMENT

None.
